# Artificial intelligence for human gunshot wound classification

**DOI:** 10.1016/j.jpi.2023.100361

**Published:** 2023-12-30

**Authors:** Jerome Cheng, Carl Schmidt, Allecia Wilson, Zixi Wang, Wei Hao, Joshua Pantanowitz, Catherine Morris, Randy Tashjian, Liron Pantanowitz

**Affiliations:** a Department of Pathology, University of Michigan, Ann Arbor, MI, USA; b Biostatistics Department, School of Public Health, University of Michigan, Ann Arbor, MI, USA; c Michigan Center for Translational Pathology, University of Michigan, Ann Arbor, MI, USA

**Keywords:** Artificial intelligence, Deep learning, Convolutional neural network, Human gunshot wound

## Abstract

Certain features are helpful in the identification of gunshot entrance and exit wounds, such as the presence of muzzle imprints, peripheral tears, stippling, bone beveling, and wound border irregularity. Some cases are less straightforward and wounds can thus pose challenges to an emergency room doctor or forensic pathologist. In recent years, deep learning has shown promise in various automated medical image classification tasks.

This study explores the feasibility of using a deep learning model to classify entry and exit gunshot wounds in digital color images. A collection of 2418 images of entrance and exit gunshot wounds were procured. Of these, 2028 entrance and 1314 exit wounds were cropped, focusing on the area around each gunshot wound. A ConvNext Tiny deep learning model was trained using the Fastai deep learning library, with a train/validation split ratio of 70/30, until a maximum validation accuracy of 92.6% was achieved. An additional 415 entrance and 293 exit wound images were collected for the test (holdout) set. The model achieved an accuracy of 87.99%, precision of 83.99%, recall of 87.71%, and F1-score 85.81% on the holdout set. Correctly classified were 88.19% of entrance wounds and 87.71% of exit wounds. The results are comparable to what a forensic pathologist can achieve without other morphologic cues. This study represents one of the first applications of artificial intelligence to the field of forensic pathology. This work demonstrates that deep learning models can discern entrance and exit gunshot wounds in digital images with high accuracy.

## Introduction

Gunshot wound (GSW) characterization is an important responsibility of emergency room physicians and forensic pathologists that may carry legal implications.^1^ When evaluating a GSW, the examining forensic pathologist has to document wound size, shape, site, and location of the wound.^2^ Wound severity is affected by several factors including the type of firearm, bullet construction, mass, velocity, and soft tissue properties.^3^ The distance of fire, point of entry/exit, and whether the wound was inflicted accidentally or self-inflicted (manner of death) are additional points that have to be determined.

Several features are helpful in the identification of gunshot entrance and exit wounds, such as the presence of muzzle imprints, soot deposition, peripheral tears, gunpowder stippling, abrasion collars, bone beveling, and wound border irregularity. At the point of bullet contact, distant range shots produce an abrasion collar due to detachment of epidermis from deeper skin layers.^4^ The width of abrasion collars are influenced by bullet design, caliber, velocity, angle, and the affected body region.^5^ Entrance wounds are usually round or oval,^4^ but show a stellate appearance when occurring over bony surfaces. Exit wounds tend to be larger, irregularly outlined, and lack gunpowder stippling and soot deposition. Some cases are less straightforward and can pose challenges to an emergency room doctor or forensic pathologist. The heterogeneity of the appearance of these wounds is a problem that is difficult to solve.

Herein, we propose using a deep learning (DL) model trained on thousands of images to assist in the classification of GSWs. To our knowledge, this is one of the first studies using artificial intelligence (AI) to help classify human GSWs. One study using AI on GSWs is a proof-of-concept study using DL and simulated gunshots on piglet carcasses to differentiate contact shots from close-range shots and distant range shots.^6^

In recent years, DL which is a type of AI, has proven to be capable of classifying images from various subject matters with high accuracy, at times even surpassing human performance.^7^ DL refers to neural networks with many layers. Convolutional neural networks (CNN) are a type of DL that are often used to solve computer vision problems such as image classification, detection, and segmentation. CNNs employ a hierarchical set of weights and processing layers to generate data representations that are capable of making predictions from digital images.^8^ Given a sufficiently large dataset, a CNN model can automatically learn to create a set of hierarchical features (e.g., curves, colors, shapes) to represent data, oftentimes enabling it to classify images with accuracies comparable to a trained pathologist.^9^ DL has been widely used for histopathological cancer identification/grading, mitosis detection, automated immunohistochemistry stain scoring, cell counting, and other types of image analysis tasks. Several DL-based tools have already attained FDA approval and/or CE Marking, including software for classifying/grading prostate cancer.^10^

DL, and CNNs in particular, have been around for several decades, but only started gaining widespread attention and usage over the past decade. The recent surge in popularity may be attributed to a number of factors, including the availability of faster computers, faster graphical processing units with more memory, large public image datasets, freely available DL architectures, and open-source tools for training DL models. Even though DL-based tools can be highly accurate, these algorithms do have some flaws, including the necessity for large amounts of training data, susceptibility to adversarial attacks,^11^ and lack of explainability, which led to its “black box” designation.

## Materials and methods

### Data preparation

A collection of 2418 digital color images (jpeg format) of entrance and exit GSWs were procured from our institution’s forensic archive. Of these, 2028 entrance and 1314 exit wounds were cropped, focusing on the area around each GSW. Some images had multiple entry/exit wounds, which led to the number of cropped images exceeding the initial number of images. Images were resized to 300 × 300 pixels in largest dimension, maintaining their aspect ratio, and augmented into 224 × 224 pixel images with varying combinations of horizontal flip, vertical flip, zoom, rotation, warp, and brightness. Several DL architectures were trained using Fastai and Jupyter Notebook on a system running Microsoft Windows 10, with a train/validation split ratio of 70/30 for 50 epochs to determine which architecture worked best with our dataset. Fastai is an open-source Pytorch-based DL framework that can provide state-of-the-art results using various DL architectures with relatively few lines of code.^12^ Computer hardware specifications included an Intel I9-9900KF CPU, 64 GB of RAM, and an RTX 3090 GPU. The ConvNext Tiny DL model performed best in the initial trial (attaining a maximum accuracy of 88.5% after 50 epochs), and the model was further trained until a maximum validation accuracy of 92.6% was achieved. Other models explored in the initial trial included popular architectures such as ResNet50, ConvNext Large, EfficientNetV2S, and MobileNetV3Large. After 50 epochs, these models achieved maximum validation accuracies of 87.5%, 87.5%, 86.9%, and 83.8%, respectively. An additional 415 entrance and 293 exit wound images were collected for the test (holdout) set. Predictions were made on the holdout set, and 100 images with the highest error rates were anonymized and distributed to 2 experienced forensic pathologists for review.

### Statistical analysis

We compared the classification rates for entrance wounds, exit wounds, and overall performance between 2 pathologists versus AI, using 100 images. To analyze the paired data between the pathologists and AI, we utilized McNemar’s test, which was applied to 3 two-dimensional contingency tables, resulting in 3 *p*-values corresponding to entrance wounds, exit wounds, and overall assessment. In addition, we plotted the receiver operating characteristic curve and calculated the area under the curve (AUC) for the holdout set using the R package “pROC”.^13^ All statistical analyses were conducted in R software (version 4.1.2).

## Results

The ConvNext Tiny model achieved an accuracy of 87.99% (95% CI 82%–94%), precision of 83.99% (95% CI 73.17%–93.33%), recall of 87.71% (95% CI 77.78%–95.83%), specificity of 88.19% (95% CI 80%–95.08%), F1-score of 85.81% (95% CI 77.65%–92.47%), and AUC of 0.946 (95% CI 0.931–0.962, Fig. 3) on the holdout set. Correctly classified were 88.19% of entrance wounds and 87.71% of exit wounds. There were 366 true entrances, 257 true exits, 49 false exits, and 36 false entrances (Table 2). The results are comparable to what a forensic pathologist can achieve without other cues.^14^

The 100 images for which the AI model had the most difficulty with classification were presented to 2 experienced forensic pathologists to assess whether they also found these images challenging. These challenging images consisted of 54 entrance wounds and 46 exit wounds. In this subsequent data subset, the AI model correctly classified 5 (9%) entrance wounds and 10 (22%) exit wounds, while our pathologists were able to correctly classify 43 (80%) entrance and 13 (28%) exit wounds. Overall, our pathologists outperformed AI (*p* < .0001), with a misclassification rate of 44% of the images for the pathologists compared to 85% for the AI (Table 1). Interestingly, the prediction accuracy between pathologists (72% misclassified) and AI (78% misclassified) for exit wounds were not found to be significantly different (*p* = .58).

**Table 1:** Prediction accuracy comparison between pathologists and AI on top-100 loss images. *p*-values were obtained from the McNemar test.

	Forensic pathologists		AI		*p*-value
Gunshot wound	Correct	Incorrect	Correct	Incorrect	
Entrance (*n* = 54)	43 (80%)	11 (20%)	5 (9%)	49 (91%)	<.0001*
Exit (*n* = 46)	13 (28%)	33 (72%)	10 (22%)	36 (78%)	.58
Total (*n* = 100)	56 (56%)	44 (44%)	15 (15%)	85 (85%)	<.0001*


Entrance GSW		AI	
		Correct	Incorrect

Pathologists	Correct	4	39
	Incorrect	1	10

Exit GSW		AI	
		Correct	Incorrect

Pathologists	Correct	5	8
	Incorrect	5	28

Total		AI	
		Correct	Incorrect

Pathologists	Correct	9	47
	Incorrect	6	38

**Table 2:** Prediction accuracy of AI on the holdout set (*n* = 708). AI prediction is considered to be “entrance” if the prediction probability for “entrance” is >= 0.5, otherwise it is “exit”.

	AI	
Gunshot wounds	Correct	Incorrect
Entrance (*n* = 415)	366 (88%)	49 (12%)
Exit (*n* = 293)	257 (88%)	36 (12%)
Total (*n* = 708)	623	85

## Discussion

**Fig. 1:**
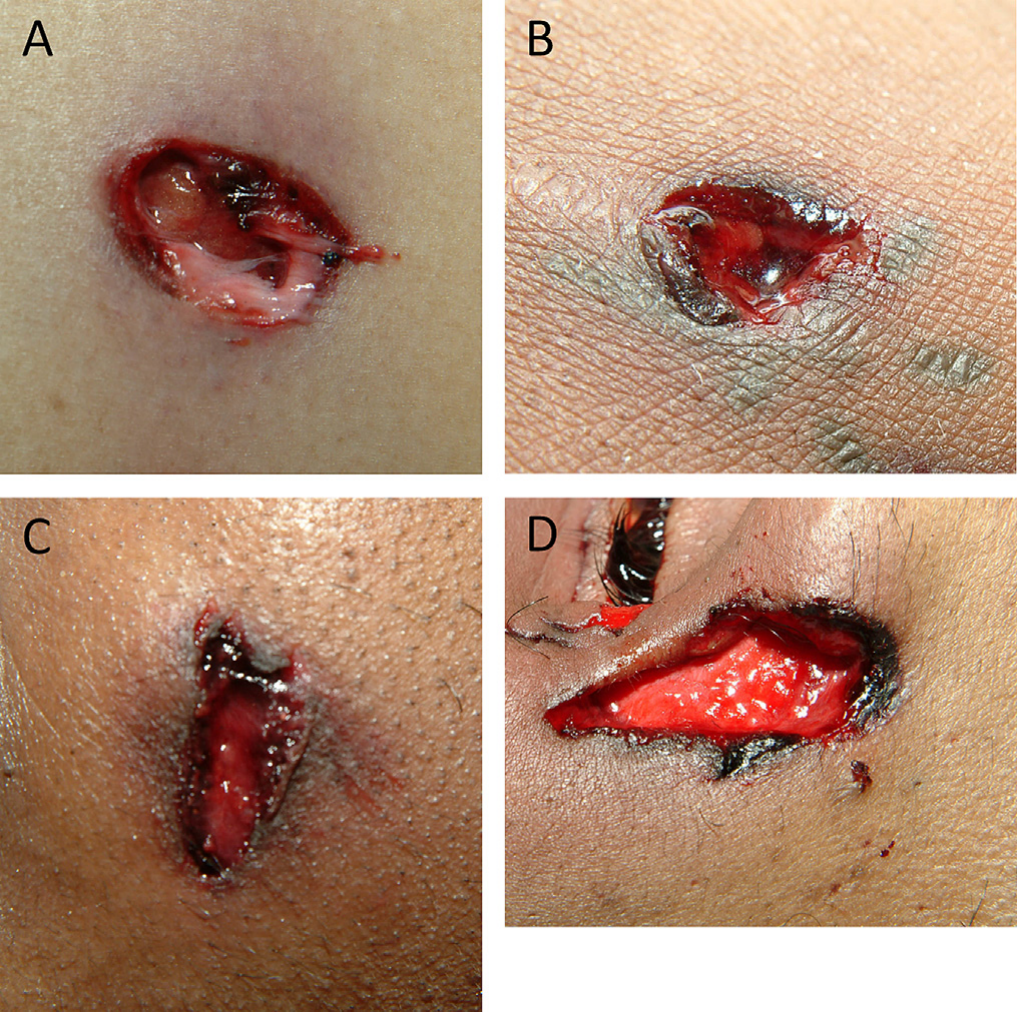
Entry wounds from holdout set misclassified by AI (A–D). Abrasion collars are a characteristic feature.

**Fig. 2:**
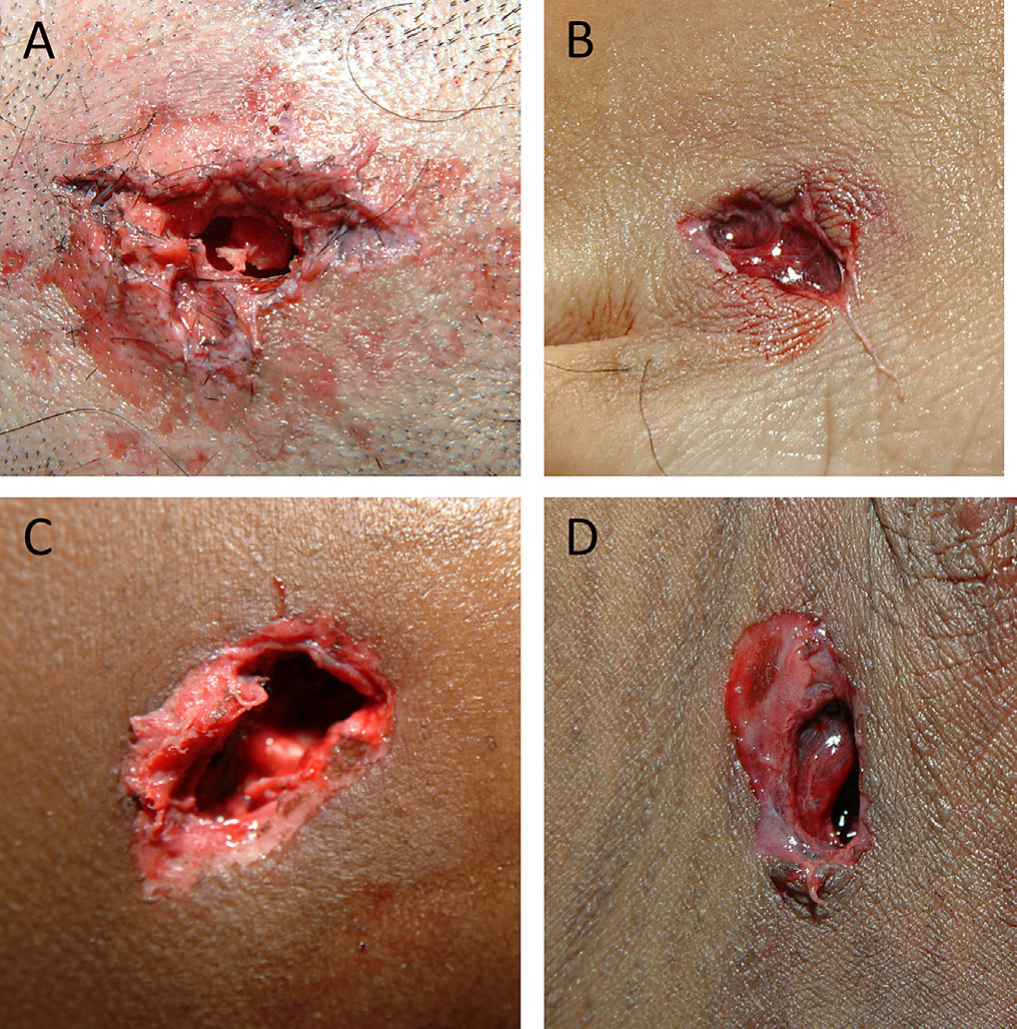
Exit wounds from holdout set misclassified by AI (A–D). Some of these wounds are difficult to classify without external cues.

Employing several comparative DL models, we were able to develop an AI-based model to correctly distinguish entrance from exit GSWs represented in digital color forensic images with high performance accuracy. Of the 100 images with the highest error rates (including 85 misclassified pictures), some were obvious entrance wounds (Fig. 1), while others were more ambiguous (Fig. 2). One case that was misclassified by our AI-based model had stippling around the entrance wound, a characteristic never present in exit wounds. One hypothetical scenario in which the AI-based model may find difficulty in the determination of entrance versus exit wounds may be in cases with multiple gunshot wounds with at least 1 retained projectile, as confirmed by post-mortem radiographs. In this situation, the AI-based model may not reliably resolve which of the wounds are entrance wounds and which are exit wounds. Another limitation of the system is the fact that image analysis was confined to 2 dimensions. Furthermore, forensic pathologists often manipulate tissues and probe GSWs with instruments in order to supplement their understanding of wound characteristics, neither of which an AI-based model is capable of replicating. Clearly, these examples indicate that there are circumstances under which wound classification cannot be performed by an AI-based model with absolute accuracy.

**Fig. 3:**
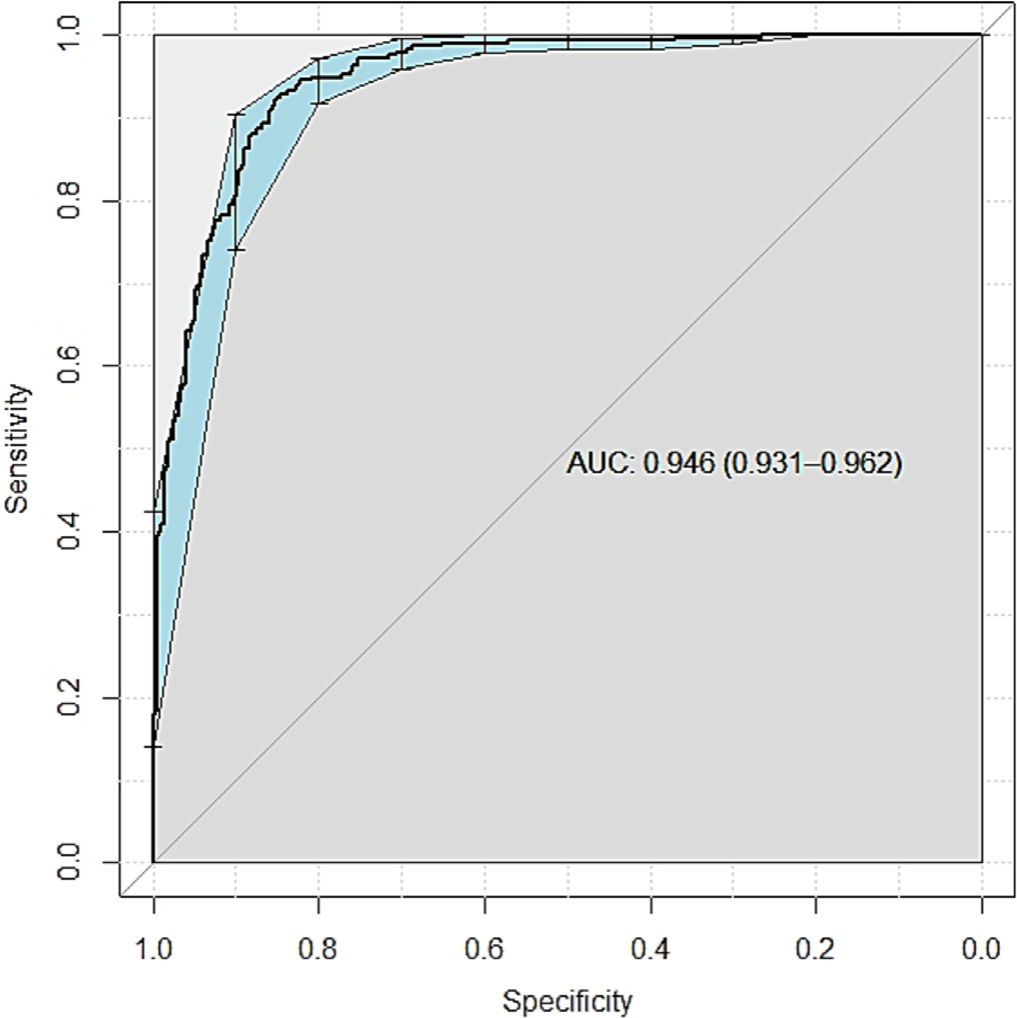
ROC curve based on AI prediction probabilities on holdout set. It had an AUC of 0.946 (95% CI 0.931–0.962).

Adding to the challenge, there are GSWs with atypical presentations that manifest under certain circumstances. For instance, entrance wounds that result from bullets that first strike an intermediary target (e.g., victims who are shot in motor vehicles) may exhibit an atypical appearance. Moreover, wounds in skin folds of obese individuals, especially when they are in contact with each other, can be a diagnostic problem. The presence of soot deposition around entrance wound margins may also be absent when a bullet passes through clothing.^15^ In addition, a close contact shotgun wound to the head can produce a stellate exit with radial tears.^16^ Cranial close range entrance wounds with radial skin tears may even resemble an exit wound.^17^

### Challenges/Limitations

In general, DL models benefit from more data, improving prediction accuracy as more training samples are added.^18^ However, image collection and preparation is very time-consuming, and thus we limited our data to 2000+ images due to effort constraints. For the images chosen, different photographers had taken these pictures which were of varying quality, leading to some underexposed pictures that may have affected model performance. Focus, exposure quality, daylight versus tungsten light, and other factors in the digital images utilized had a role in the final picture quality. Our dataset contained more entrance wounds than there were exit wounds, and it is plausible that there were not enough examples for every condition. Radial tears in contact entrance wounds in skin overlying bone (like the skull) may impart a stellate appearance, which our DL model may have difficulty in differentiating from stellate-appearing exit wounds without being trained with a larger number of similar samples.

## Conclusions

This study represents one of the first applications of AI to the field of forensic pathology. This work demonstrates that it is feasible to train a DL model to classify entrance and exit GSWs in digital images with reasonable accuracy. In challenging situations where AI is unable to reliably classify a GSW, additional information, including the circumstances of the case, radiographs, and the ability to manipulate the body itself are essential to derive a more definite assessment.

## Declaration of Competing Interest

The authors declare the following financial interests/personal relationships which may be considered as potential competing interests:

L. Pantanowitz is on the scientific advisory board for Ibex and NTP, serves as a consultant for AIxMed and Hamamatsu and is a co-owner of Placenta AI and LeanAP. Editorial board of Journal of Pathology Informatics - L.P., J.C. If there are other authors, they declare that they have no known competing financial interests or personal relationships that could have appeared to influence the work reported in this paper.
